# Addressing concerns of access and distribution of health workforce: a discrete choice experiment to develop rural attraction and retention strategies in southwestern Ethiopia

**DOI:** 10.1186/s12913-024-11971-4

**Published:** 2024-12-18

**Authors:** Abdela Alite Hilo, John McPeak

**Affiliations:** 1 College of Health Sciences, Jinka University, Jinka, Ethiopia; 2 Department of Public Administration and International Affairs, Maxwell School of Citizenship and Public Affairs, Syracuse University, Syracuse, NY United States of America

**Keywords:** Discrete choice experiment, Human resources for health, Rural areas, Health workers, South Omo zone, Ethiopia

## Abstract

**Background:**

There is a shortage of health workers in Ethiopia, with an uneven distribution between urban and rural areas. To formulate effective policy interventions aimed at attracting and retaining health workers in rural regions, this study examined the stated preferences of health workers when selecting health jobs.

**Methods:**

A discrete choice experiment was conducted among health workers in the Aari and South Omo zones of the South Ethiopia region, from September to November 2022 to gather insights into their job preferences. The design of the discrete choice experiment was informed by literature review, focus group discussions, and in-depth interviews. Through these qualitative studies, key job attributes influencing health workers’ preferences were identified, including salary, education, housing, location, timeliness of payment, medicine and equipment, management culture, and infrastructure. We employed a mixed logit model, allowing for full correlation between utility coefficients, to assess the relative importance of these attributes and account for heterogeneity in preferences and scales. We also conducted a willingness-to-pay analysis and assessed the probability of job uptake for both single and combined incentives.

**Results:**

All eight attributes exhibited statistically significant effects, with the expected signs, and indicating preference heterogeneity. Education opportunity, salary, and housing emerged as the most influential attributes shaping health workers’ decisions when considering a rural posting as a future workplace. Notably, health workers were willing to trade off a significant portion of their salary for improvements in other aspects of the job. Subgroup analysis revealed that health workers without a rural background were willing to pay more to work at the relatively more urban zone center compared to those with rural experience. Offering educational opportunities after one year of service at the base salary increased the probability of choosing a rural job posting by 79.8%. In addition, we find that packages of incentives are usually preferred over a single incentive.

**Conclusion:**

Health workers express a willingness to relocate to or continue serving in rural areas contingent upon improved working conditions. Both monetary and non-monetary policy interventions should be considered by policymakers to attract and retain health workers at rural locations in southwestern Ethiopia.

**Supplementary Information:**

The online version contains supplementary material available at 10.1186/s12913-024-11971-4.

## Background

The health workforce is a fundamental component of the World Health Organization’s (WHO) health systems framework and is critical for achieving universal health coverage [1, 2]. While adequate staffing levels are associated with improved maternal and child health outcomes, only half of the global population has access to the health workforce necessary for quality care [3, 4]. According to WHO, 57 developing nations, including Ethiopia, face severe shortages of healthcare personnel [5]. Despite global efforts to expand the health workforce, unequal distribution persists between developed and developing nations and within urban and rural areas, limiting access to healthcare for rural residents in many developing countries. This inequality underscores the urgent need for more equitable allocation of health workers globally [2, 6, 7].

WHO recommends several strategies to increase and equitably distribute the health workforce, including offering post-service education, ensuring better working conditions, and recruiting students with rural backgrounds [7]. Ethiopia has adopted these WHO recommended policies and invested significantly in its health sector over the past two decades. As a result, the ratio of health workers to the population rose from 0.97 per 1000 people in 2013 to 1.63 per 1000 in 2016 [6]. Despite these improvements, Ethiopia’s health worker-to-population ratio remains among the lowest globally, still below the WHO’s 2025 target of 2.3 for sub-Saharan Africa [8]. Furthermore, health workers are unevenly distributed across Ethiopia’s urban and rural areas. Although an estimated 77% of Ethiopia’s population lives in rural areas [9], most health infrastructure and health workers are concentrated in urban areas [10].

This shortage and uneven distribution of health workers lead to significant access issues for maternal and child health services. Nationally, only 26% of childbirths occur in health facilities, and this percentage drops to 15% in Afar, a region where 81% of the population resides in rural areas, in stark contrast to the entirely urban population of Addis Ababa, where 97% of childbirths occur in health facilities. The national under-five mortality rate stands at 67 deaths per 1000 live births, but these rates vary widely — ranging from 39 deaths per 1000 live births in Addis Ababa to 125 deaths per 1000 live births in Afar [11].

The Ethiopian government is committed to reducing healthcare access disparities by implementing strategies to attract and retain healthcare professionals in regions where a large share of the population lives in rural areas. Despite these efforts, retaining staff in these regions remains challenging [8]. Developing effective incentive schemes hinges on understanding factors that influence health workers' job location decisions. However, there is limited research on human resources for health in Ethiopian. Local policymakers require evidence from robust research to understand factors influencing health workers’ decisions within their specific contexts and identify actionable steps that can create an impact. Recently, discrete choice experiments (DCEs) have gained traction for providing quantitative insights into the relative importance of job attributes influencing health workers’ location choices, as well as illustrate the trade-offs among these factors [12–15].

Studies in developed countries using DCEs have shown that both monetary and non-monetary incentives significantly influence health workers’ decisions to work in rural areas. Research in Australia found that a 64% salary increase and strong social interactions were key factors influencing doctors to serve in these locations [16]. Similarly, a stated preference study in Germany identified that monetary incentives, such as pay raises, and non-monetary incentives, like onsite childcare, were important in attracting health workers to rural areas [17].

DCE studies in low- and middle-income countries have also identified various incentives that attract health workers to rural settings. A multi-country study highlighted that monetary incentives, such as rural allowances and improved health coverage, along with non-monetary incentives like opportunities for further education, are key attributes that attract health workers to rural health facilities [12]. Additional studies in these settings consistently report that both monetary and non-monetary factors impact health workers’ decisions to relocate to or remain in rural health facilities [18–22].

Studies using DCEs show that increasing salary generally improves health workers’ willingness to work in rural areas, although the strength of this effect varies across studies [18, 20, 23]. Non-monetary incentives, such as housing, facility quality, and supportive management culture, have also been shown to play crucial roles in recruitment and retention in rural locations [24]. A recent study identified that opportunities for educational advancement and adequate workplace infrastructure are particularly important in health workers’ decisions regarding job location [20]. Additionally, factors such as equipment availability, medication supply, and facility location further affect health professionals’ choices [15, 25].

Recent advances in analyzing DCE data emphasize accounting for preference heterogeneity based on sociodemographic characteristics [25, 26]. Variables such as gender, age, ethnicity, education level, family income, and rural upbringing significantly influence willingness to work in rural areas [20]. Notably, individuals from rural backgrounds respond differently to monetary incentives; while some studies suggest they are more sensitive to salary increases, others find that they are less responsive to monetary incentives than their urban counterparts [20, 25]. These findings emphasize the importance of considering individual characteristics when examining motivations to work in rural settings.

In Ethiopia, both qualitative and quantitative studies have found that both wage and non-wage factors influence health workers’ location choices [27–30]. For example, a qualitative study by Ayalew and colleagues identified that higher salaries, opportunities for professional growth, and recognition are key motivators for health workers [27]. Similarly, a systematic review and meta-analysis highlighted that opportunities for professional growth and strong staff relationships enhance staff job satisfaction and motivation in Ethiopia [29]. DCEs conducted at national and state levels in Ethiopia revealed that higher salary, quality housing, education upgrading opportunities, availability of medicine and equipment, and supportive work environment are crucial job characteristics that attract health workers to rural areas [28, 30].

While much of the literature relies on national-level data to infer health workers’ preferences across different contexts, there is growing recognition of the importance of local data for informing incentive policies, particularly in rural areas of developing nations [22, 30, 31]. Understanding the effectiveness of various policy options in encouraging health workers to accept and remain in postings to areas where a large share of the population is rural requires tailored study [7, 22, 30, 32]. This research builds upon previous studies by examining subnational data to explore health workers’ job preferences. It considers both preference and scale heterogeneity, explores interactions between random coefficients, examines their interactions with sociodemographic variables, and adds valuable insights to the literature on health workers’ job preferences [26, 33].

In this study, we investigate the preferences for job attributes among health workers in South Omo zone in southwestern Ethiopia. A DCE was conducted among health workers in the former South Omo zone of the current South Ethiopia Regional State. This largely rural zone, with only 7.5% of the population living in urban areas (2007 Census), was selected by the lead author, who has both professional and personal connections to the area. Achieving a more equitable distribution of the health workforce is a key health policy agenda that requires government commitment to ensuring decent working conditions in rural settings [7]. This study of decision making among health workers in the former South Omo zone contributes to this agenda by examining factors affecting their location preferences.

## Data and methods

### Setting and timing

This study was conducted in the South Omo zone, which was part of the former Southern Nation Nationalities and Peoples Regional State (SNNPR) and the current South Ethiopia Regional State, targeting health workers currently working in public health facilities from September to November 2022. Located in southwestern Ethiopia, bordering northern Kenya and the southeastern South Sudan, the South Omo zone is one of the most remote areas in the country, primarily inhabited by pastoralist and agropastoral communities. The relatively urbanized zone center, Jinka, is approximately 750 km from the capital Addis Ababa, accessible by paved road, which typically requires a 12-hour journey by bus. In contrast, the 200 km gravel road from Hadho in the Dassenech district to Jinka takes around 7 h, highlighting the challenging travel conditions on unpaved routes in the region.

The South Omo zone experiences persistent shortages of qualified health workers due to its remoteness and the limited number of skilled health workers native to the area. Because of high illiteracy rates among most indigenous communities, the public sector is largely staffed by workers from Ethiopia’s central and highland regions. Health workers from these areas sometimes refuse rural postings in South Omo or depart as soon as they find work elsewhere, leading to high turnover and staffing shortage. As a result, health facilities in the zone are usually understaffed or staffed with inexperienced workers (personal interview with the South Omo Zone Human Resources Director).

### Design of the discrete choice experiment

A discrete choice experiment (DCE) is a quantitative method used to evaluate different factors influencing job choices. DCEs are widely used in fields such as health economics, human resources for health, and transportation [22, 34, 35]. The method is grounded in random utility theory and consumer choice theory, relying on the assumption of economic rationality and utility maximization [35, 36]. According to Lancaster’s consumer theory, DCEs assume that individuals’ choices are influenced by the attributes of a good or service rather than the good itself [37].

DCEs are regularly utilized in the human resources for health literature to elicit the employment preferences of health personnel, as job choices are influenced by specific job attributes [18, 30]. Unlike qualitative surveys that list factors influencing job choices, DCEs provide quantitative information on the relative importance of these attributes, trade-offs between them, and the probability of health personnel accepting a job with varying attribute levels [38]. This method presents health workers with hypothetical job scenarios, each varying in attribute levels, to elicit preferences. An individual’s choice indicates preferences towards specific attributes or their combinations [39]. DCEs are particularly useful due to the challenges of gathering real data on non-existent job positions and the general lack of human resource data in the health sectors of developing nations [40]. Notably, studies have shown that DCE findings can effectively predict actual behavior within the public health sector [41].

A DCE involves three main components: an experimental design to create a choice survey and generate choice data; analysis to estimate preferences from the choice data; and the application of these estimates to derive willingness to pay, and job uptake rates [42]. We employed a generic choice approach, where job labels (e.g., Job A or Job B) are inherently meaningless, and only a combination of attribute levels gives meaning. This approach aligns with our goal of evaluating trade-offs among different attributes of healthcare jobs in rural areas [33]. The main stages of the DCE process include identifying attributes and levels, designing the experiment, collecting data, inputting data, and analyzing and interpreting the results [42].

### Defining attributes and levels

The first stage involved identifying key attributes and their respective levels. Attributes relevant to rural healthcare jobs were prioritized. A literature review on factors influencing the job preferences of health workers in low- and middle-income countries identified thirteen key attributes: improved salary, opportunities for education upgrading, hardship allowances, short-term training opportunities, length of compulsory service time, presence of a private wing, housing provision, proximity to urban centers, degree of insecurity in the surrounding area, quality of education for children, quality of management at the health center, availability of medicine and equipment, and infrastructure, including electricity and other amenities.

In the second stage, 18 in-depth interviews with policymakers and healthcare professionals were conducted to identify feasible attributes for policy implementation. In the third stage, seven focus group discussions were held with zone health department management members, district health officers, health center managers, and healthcare workers to assess and rank the compiled attributes. Following these three steps, additional phone conversations with health workers identified timeliness of payment as a critical attribute. Payment delays had become of increasing importance during the time the field work was conducted due to budget shortages arising from domestic conflict [43]. For example, in Maale District, which is another area with a large rural population, all physicians left their posts after a year-long payment delay (personal communication, July 15, 2022).

The literature review, focus group discussions, and in-depth interviews resulted in a final list of eight attributes (Table 1). These attributes encompass a range of factors and aligns with those specified in other DCEs, including gross monthly salary [18, 21, 22], opportunity for education upgrading [14, 20, 22, 44], housing [18, 22, 24], location [12, 30], workplace (management) culture [18, 28], infrastructure [18, 20, 24], and the availability of equipment and medicine [20, 30]. Timeliness of payments, as discussed, is a unique attribute in this study, added due to the current economic situation in Ethiopia.

Attribute levels were determined through focus group discussions and in-depth interviews that were described above, where participants were asked to suggest realistic options for each attribute. For instance, participants identified feasible housing levels for rural deployment and salary levels that the Ethiopian government could reasonably be able to pay. Each attribute was then divided into levels and incorporated into the questionnaire, as shown in Table 1.

**Table 1:** DCE Attributes and Levels for Health Workers in South Omo, Ethiopia

Attributes	Levels	Description
Salary	1	Your base salary at its current level
	2	Your base salary at its current level + 40% remoteness bonus
	3	Your base salary at its current level + 60% remoteness bonus
	4	Your base salary at its current level + 80% remoteness bonus
Education upgrading opportunity (with full tuition and continuous payment of salary)	1	No education upgrading opportunity is offered
	2	Education opportunity is offered after three years of service
	3	Education opportunity is offered after two years of service
	4	Education opportunity is offered after one year of service
Provision of housing	1	No housing is provided by the health facility
	2	Basic housing (studio with shared kitchen and shared toilet) is provided by the health facility
	3	Advanced housing (two rooms with private kitchen and toilet) is provided by the facility
Location of the health facility	1	Health facility is two hours away from district center by car
	2	Health facility is located at district center or towns of same level
	3	Health facility is located at zonal center (Jinka)
Timely payment of salary and benefits	1	Salary and other benefits delayed by more than two months.
	2	Salary and other benefits delayed by a month
	3	Salary and other benefits paid on time
Workplace management and culture	1	Supervisors are not supportive and make work difficult.
	2	Supervisors are supportive and make work easy
Availability of equipment and drugs at the health facility	1	Inadequate (basic equipment’s and medicine stock not available at least for two months)
	2	Adequate (basic equipment’s and medicine stock available at least for two months)
Infrastructure (electricity, water, transportation, & internet)	1	Below average access to infrastructure (electricity, internet, water, and transportation)
	2	Average or above average access to infrastructure (electricity, internet, water, and transportation)

### Experimental design

In the second stage, after defining the attributes and attribute levels, hypothetical job scenarios were generated by combining these elements. Among the eight DCE attributes, two had four levels, three had three levels, and two had two levels. A full-factorial design would generate 3456 (4^2^ × 3^3^ × 2^3^) possible scenarios. With each choice set containing two options, the total number of unique choice sets would be (3456 × 3455)/2, resulting in 5,970,240 – a number impractical to present and unrealistic for respondents to process. To address this issue, a D-efficient fractional factorial design was developed (assuming zero priors) using Sawtooth Software’s Lighthouse Studio 9.14.2. This design employed a full-profile, conjoint-based choice approach with balanced overlap to minimize the number of choices presented to respondents [45, 46].

**Fig. 1:**
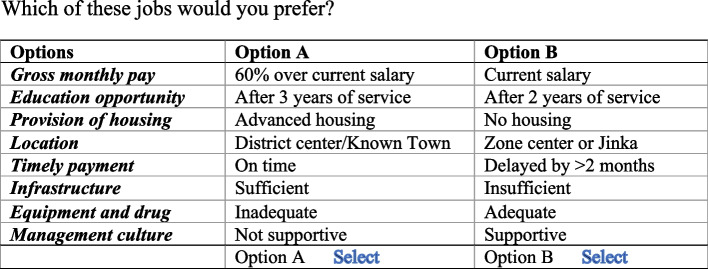
Example choice set: health workers

The design was assessed to ensure that it met efficient design criteria, including orthogonality, level balance, and minimal overlap. Sixteen choice sets, each with two options, were created. The respondents were presented with these 16 choice scenarios, as shown in Fig. 1, where they had to choose between option A and option B in each choice scenario without an option to express indifference or choose neither.

### Sample size determination

The sample size was determined based on feasibility, considering both financial and time constraints. All individuals present at the health facility during the data collection team’s visit^1^were invited to complete the survey if they consented. A total of 314 health workers across 25 health centers completed the survey. This sample size exceeds the typical recommendations for discrete choice experiments (DCEs), including those suggested by Hensher et al. using the rule of thumb [47], the minimum sample size approach by Pearman et al. [48], or sample size calculation method as proposed by Louviere et al. [45].

### Survey and data collection

In addition to the DCE, the questionnaire included questions about respondents’ sociodemographic characteristics and career and college experiences. The survey was conducted between September and November 2022 in health center meeting halls, with participants completing the survey independently in the presence of the research team using computers, tablets, and phones with mobile internet. The survey link was distributed via messaging apps, with completion taking 30 to 45 min on average. Responses were stored directly in Sawtooth Lighthouse’s data portal. A total of 314 participants generated 10,048 observations. The data were transferred from Lighthouse Studio to Excel and then imported into STATA for cleaning and analysis.

The survey’s purpose and DCE instructions were explained in detail at each health center by the lead author in Amharic. Informed electronic consent was obtained from all participants, each of whom was provided with a comprehensive statement explaining the study’s voluntary nature and assuring the confidentiality of their responses. Ethical approval (IRB No. 22–109) was obtained from the Ethics Review Board of the Graduate School of Syracuse University prior to the fieldwork. Additionally, consent to conduct the study was obtained from the research department of the state government of the SNNPR.

### Empirical methods

The random utility model serves as the theoretical foundation for analyzing DCE data, assuming that consumers are rational and aim to maximize their utility by selecting from defined options [35]. To estimate respondents’ preferences for job attributes, we employed a mixed logit model. This model relaxes the restrictive assumptions of the basic multinomial logit model and accounts for heterogeneity in preferences across respondents and choice sets. According to Hess and Train (2017), a mixed logit model with full correlation among utility coefficients also accounts for other sources of correlations, including those from scale heterogeneity [26]. The utility function is specified as follows:

$$\:U_{isj}={X'\:}_{isj}{\beta\:}_i+{\epsilon\:}_{isj\;}\;i=1,...,\;N;\;j=1,...,\;J;\;s=1,...,S$$ (1).

$\:{U}_{isj}$ is the utility that individual *i* derives from choosing alternative j in choice scenario s, X is a vector of observed attributes and corresponding levels, $\:{\beta\:}_{i}$ is a parameter to be estimated reflecting the desirability of the attributes and varies over people, and $\:{\epsilon\:}_{isj}$ is a random term that represents the unobserved component of the utility [49]. In addition to the mixed logit model with full correlation between utility coefficients, we used other econometric approaches to estimate the utility functions, including the conditional logit model and mixed logit model, where the utility coefficients were not correlated. These models were compared using the Akaike information criterion (AIC) and Bayesian information criterion (BIC), and a mixed logit model with full correlation between utility coefficients was the best model for the data [42].

The probability that an individual will choose a particular job is modeled by indirect utility based on the attribute and attribute levels of the choice experiment:

*V = ß*_*1*_*Salary + ß*_*2*_*Edu*_*_*_*3 + ß*_*3*_*Edu*_*_*_*2 + ß*_*4*_*Edu*_*_*_*1 + ß*_*5*_*Loc_dist + ß*_*6*_*Loc_zn + ß*_*7*_*Housing_basic + ß*_*8*_*Housing_advanced + ß*_*9*_*Mnth_delay + ß*_*10*_*Pmnt_ontime + ß*_*11*_*Equipdrugs_adeq + ß*_*12*_*Infra_suff + ß*_*13*_*Mgt_supp + ß*_*14*_*Const* + $\:{\epsilon\:}_{isj}$ (2).

All attributes were dummy coded and specified as having a random component, except for the salary, which was coded as a continuous variable in the model to facilitate the calculation of willingness to pay (WTP). We estimated utility coefficients using Stata’s mixlogit user-written command [50]. Due to the non-closed form, the model was estimated using simulated maximum likelihood with 500 Halton draws [35].

Willingness to pay (WTP) is the relative monetary value health workers place on attributes of job options. We obtained the willingness to pay (WTP) by calculating the ratios of the coefficients between each attribute level and the monthly salary attribute. A positive WTP result indicates the amount of monetary value participants are willing to pay to obtain a desirable attribute level, and a negative value indicates the amount participants are willing to be compensated to accept an undesirable attribute level. We calculated the 95% confidence intervals for the WTP using the delta method [51]. We also carried out a simulation study to predict the percentage of health workers who would prefer a job posting that presented a package of incentives over other available jobs without those benefits, using the following equation based on output from mixed logit analysis model:

3 $$\:\:\:\:\:\:\:\:\:\:\:\:\:\:\:\:\text{I}\text{n}\text{c}\text{e}\text{n}\text{t}\text{i}\text{v}\text{e}\:\text{P}\text{a}\text{c}\text{k}\text{a}\text{g}\text{e}\:=\:\frac{\left\{\frac{{e}^{\left(proposed\:preference\:value\right)}}{{e}^{\left(baseline\:preference\:value\right)}}\right\}}{\left\{1+\left\{\frac{{e}^{\left(proposed\:preference\:value\right)}}{{e}^{\left(baseline\:preference\:value\right)}}\right\}\right\}}$$

The baseline preference values represent the baseline level where provided packages consist of the worst possible set of attribute values as incentives. The proposed preference values are obtained when one or more attribute levels are changed from the baseline to a higher level [44]. Furthermore, we conducted sub-group analysis to determine similarities or differences in preferences among different sub-groups. This is done to determine if there are strategies for health worker recruitment and retention that are more likely to succeed with sub-groups.

## Results

### Descriptive statistics

The demographic and work related characteristics of the 314 health workers who participated in the survey are presented in Table 2. The mean age of the survey participants was 26.91 years (SD = 4.02), and the average monthly salary among participants was 6,093 ETB (SD = 1,575 ETB). Most participants (72.9%) were male, while female accounted for 27.1%. Regarding ethnicity, 58% of respondents belonged to non-indigenous groups in the South Omo zone, whereas 42% were from indigenous groups. The majority of participants (62.1%) were unmarried, and for those with children, the average number of children was 1.08 (SD = 1.65). In terms of education, 42.4% held bachelor’s degrees or higher, while 57.6% had associate degrees. The majority (70.1%) had four years or fewer work experience, while 29.9% had more than four years. Additionally, 77.1% of participants had prior rural work experience, compared to 22.9% who did not. Most respondents (76.4%) reported a rural background, having lived in a rural area for at least part of their childhood. When asked about their willingness to work in rural health facilities, 33.8% indicated they were extremely likely, 45.5% somewhat likely, 9.9% somewhat unlikely, and 10.8% extremely unlikely to do so. When considering the condition of rural job postings, 84.7% said they would accept a rural job if conditions were decent, while 15.3% said they would not.

**Table 2:** Descriptive statistics of health workers, South Omo Zone, SNNPR, Ethiopia, 2022

Respondent characteristics	(*N*)	(%)
**Age**,** mean years (SD)**	314	26.91 (4.02)
**Gender**		
Female	85	27.1
Male	229	72.9
**Ethnicity**		
Indigenous to South Omo	132	42
Non-indigenous to South Omo	182	58
**Marital status**		
Currently married	119	37.9
Currently not married	195	62.1
**Rural background**		
No	74	23.6
Yes	240	76.4
**No of children**,** mean (SD)**	314	1.08 (1.65)
**Years of work experience**		
Less than or equal to four years	220	70.1
Greater than four years	94	29.9
**Education Level**		
Associate degree	181	57.6
Bachelor’s and above	133	42.4
**Rural Work Experience**		
No	72	22.9
Yes	242	77.1
**Will you work in rural health**		
Extremely likely	106	33.8
Somewhat likely	143	45.5
Somewhat unlikely	31	9.9
Extremely unlikely	34	10.8
**Accept Job if Condition is Decent**		
No	48	15.3
Yes	266	84.7
**Salary (ETB: 1USD = 53.4)**,** mean (SD)**	314	6093.12 (1575)

**Fig. 2:**
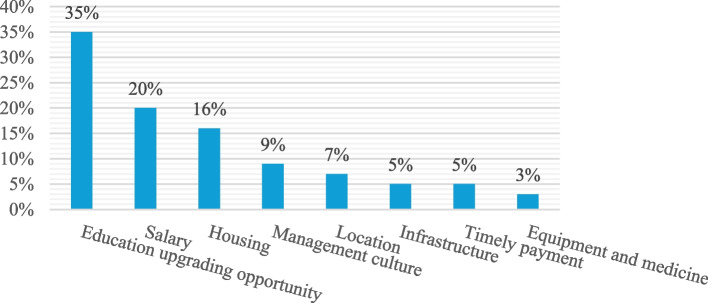
Figure that demonstrates the importance of healthcare job attributes

The relative importance of various attributes in shaping the job preferences of health workers in the former South Omo zone based on data from the DCE survey is presented in Fig. 2. The importance of each attribute was calculated by the logit function in Sawtooth Software Lighthouse Studio 9.14.2. Figure. 2 shows that, on average, the opportunity for education upgrading has the greatest influence on health workers’ choices, followed by the gross monthly salary and the provision of housing.

### Discrete choice experiment estimates

#### Job preferences among health workers in the South Omo zone

Table 3 presents the main results from the mixed logit model. The analysis revealed that the mean coefficients of at least one level of each attribute were statistically significant and exhibited the expected sign. All statistically significant coefficients were positive, indicating a positive impact on the uptake of a hypothetical healthcare job. This outcome aligns with our definition of the baseline attribute set as the worst possible value for each attribute. Among the eight attributes, health workers expressed the strongest stated preference for jobs offering education upgrading opportunities after one year of service (β = 1.414, *p* < 0.01). The provision of advanced housing (β = 0.6274, *p* < 0.01) and supportive management culture at health facilities (β = 0.349, *p* < 0.001) followed as the next most preferred attributes. The coefficient on salary indicated that respondents preferred healthcare jobs with higher salaries, all else being equal. Additionally, the availability of adequate medicine and medical supplies (β = 0.121, *p* < 0.05), sufficient infrastructure (β = 0.170, *p* < 0.01), and prompt payment (β = 0.213, *p* < 0.01) positively affected health workers’ job choices, although their impact was relatively smaller compared to education upgrading opportunities.

**Table 3:** Results of a mixed Logit Model of DCE Data from Health Workers

Attribute Levels	($\:\varvec{\beta\:}$)	Standard error	95% confidence interval	
Salary (percent increase)	0.0093***	0.0009	0.075	0.0111
Education upgrading opportunity (ref. no opportunity)				
Education after 3 years of service	0.758***	0.0911	0.579	0.936
Education after 2 years of service	1.155***	0.1007	0.958	1.135
Education after 1 year of service	1.414***	0.1213	1.176	1.651
Provision of housing *(ref. no housing)*				
Provision of basic housing	0.5007***	0.0798	0.344	0.657
Provision of advanced housing	0.6274***	0.0799	0.471	0.784
Location (ref. 2 h away from district center)				
Health facility at district center	0.114*	0.0682	−0.0198	0.247
Health facility at zonal center	0.289***	0.073	0.145	0.433
Payment timeliness (ref. delay by > 2months)				
Payment delays by a month	0.0233	0.067	−0.108	0.155
Payment delivered on time	0.213***	0.071	0.074	0.352
Management culture (ref. not supportive)				
Management is supportive	0.349***	0.056	0.239	0.460
Equipment and medicine (ref. inadequate)				
Adequate	0.121**	0.048	0.027	0.216
Infrastructure (ref. insufficient)				
Sufficient	0.170***	0.049	0.074	0.266
Log-likelihood	−3029.9196			
AIC	6087.8392			
No. Respondents	314			
No. Observation	10,048			

*P < 0.10, **P < 0.05, ***P < 0

Note: The coefficients (β) signify the average relative utility of each attribute, considering other attributes in a choice set; larger values indicate greater utility and preference for the respective attributes; *AIC* Akaike information criterion, The observations are the number of respondents multiplied by 16 choice sets multiplied by 2 options per choice set

#### Estimated willingness to pay for job attributes

Table 4 shows the results of the Willingness to Pay (WTP) calculation, indicating the percentage of monthly salary that health workers were willing to forgo for improvements in other job attributes. Health workers were willing to pay 81.35% of their monthly income for jobs that provide education upgrading opportunities after three years of service, compared to jobs without such opportunities. When the waiting period for education opportunities was reduced to one year, respondents were willing to sacrifice over 151.84%^2^of their monthly salary. Both focus group discussions and in-depth interviews revealed that health workers had a strong desire to advance their education, motivated by the potential for higher earnings and opportunities in the non-governmental sectors.

**Table 4:** Willingness to pay estimates (% of salary in ETB)^a^

Attribute levels	WTP (%)	Lower Level of 95% Conf. Interval	Upper Level of 95% Conf. Interval
Education opportunity (ref. no opportunity)			
Education after 3 years	81.35***	57.61	105.09
Education after 2 years	124.03***	94.03	154.03
Education after 1 year	151.84***	115.38	188.29
Provision of housing (ref. no housing)			
Basic housing	53.77***	34.71	72.83
Advanced housing	67.38***	47.11	87.66
Timely Payment (ref. delay > 2months)			
Delays by a month	2.50	−11.62	16.62
On time	22.90***	7.46	38.35
Location (ref. 2 h away from district center)			
District center	12.22*	−2.29	26.72
Zone center	31.03***	14.68	47.38
Management culture (ref. not supportive)			
Supportive	37.52***	24.07	50.98
Availability of equipment and drugs (ref. inadequate)			
Adequate	13.05**	2.74	23.35
Infrastructure (ref. insufficient)			
Sufficient	18.27***	7.54	28.99
Log-likelihood	−3029.9196		
No. of respondents	314		
No. of observations	10,048		

*P < 0.10, **P < 0.05, ***P < 0.01

^a^1000 ETB = 18.44 USD (November 2022) National Bank of Ethiopia

In terms of housing, respondents were willing to forgo approximately 54% of their monthly salary for basic housing provision, with this figure increasing to approximately 67% for an advanced housing option. Additionally, about 31% salary increase would be necessary to incentivize them to work in a rural area over a relatively more urbanized zonal center. Respondents also indicated a willingness to reduce their monthly salary by about 38% to work in a health facility with supportive management rather than in a facility with a challenging management environment. On average, the participants were willing to forgo about 18% of their monthly salary to work in an area with sufficient infrastructure^3^and about 13% to work in a facility with continued availability of essential medicine and equipment. However, the percentage of salary they were willing to forgo for these factors was relatively lower.

#### Subgroup analysis

Table 5 presents the WTP results among different subgroups of health workers. Except for health workers without a rural background, other subgroups exhibited relatively similar preferences for job attributes. The WTP of participants with no rural background deviated significantly from other groups except regarding management culture. Participants who had never lived in rural areas for more than one year highly valued working at zonal centers, with a WTP of 68% of their monthly salary (95% CI, 45.1–91.2), compared to health workers who had lived in rural areas for over a year, who were WTP only 22.3% (95% CI, 3.4–41). Health workers with an associate degree highly valued education opportunities after one year of service, with a WTP of 181% of their monthly salary (95% CI, 107–255), surpassing participants with a bachelor’s degree, who were WTP 120% of their monthly salary (95% CI, 82.3–158).

While both groups valued education, associate degree holders demonstrated greater interest, likely driven by the anticipation of future salary increases and enhanced self-esteem from upgrading their qualifications. Additionally, associate degree holders placed a high value on advanced housing, showing a WTP of 90% of their monthly salary (95% CI, 49.6–131.6), in contrast to bachelor’s degree holders, who were willing to pay only 49% (95% CI, 29.4–68.3).

**Table 5:** Results WTP (percentage of salary) for subgroups; 95% confidence interval

Attribute Levels	Rural Background	No background	Associate degree	Bachelor’s Degree
Education opportunity (ref. no opportunity)				
Education after 3 years	92.3(63–121.6)	29.2(1.4–57)	92.3(47.3–137)	68.1(44.2–92)
Education after 2 years	135.6(98.4–172.8)	80.2(51–109-2)	152.9(92.5–213)	96.8(66.6–126.8)
Education after 1 year	171.2(124–218.4)	92.2(59.7–124.6)	181.4(107–255)	120(82.3–158)
Provision of housing (no. housing)				
Basic housing	64.6(40.4–88.9)	14.6(−5.4-34.6)	69.9(31.4–108)	36.8(18.6–55)
Advanced housing	79(52.9–104.7)	43.5(20.1–66.8)	90.3(49–131-6)	49(29.4–68.3)
Location (ref. 2 h away from district center)				
District center	16.5(−1.3-34.2)	−5.6(−23.4-12.2)	−0.8(−29.6-28)	20(5.4–34.3)
Zone center	22.3(3.4–41)	68.2(45.1–91)	30.8(0.86–60.7)	35(17.4–52)
Payment timeliness (ref. delay by > 2months)				
Delay by a month	6.7(−11.6-24.9)	−16.9(−34.8-0.9)	1.3(−30-32.5)	1.3(−12.8-15.4)
On time	26.8(7.2–46.4)	−10.1(−30.5-10.3)	20.8(−6.5-48.2)	21(4.1–37.8)
Management culture (ref. not supportive)				
Supportive	36.7(20.9–52.6)	42.1(25.5–58.6)	41.2(17.3–65.2)	35(20.5–49.5)
Equipment and medicine (ref. inadequate)				
Adequate	15.1(2.4–27.8)	3.1(−11-17.5)	14.7(−5.6-35)	11(0.3–21.6)
Infrastructure (ref. insufficient)				
Sufficient	23.3(10–36.6)	4.1(−8.6-16.8)	16.7(−2.4-35.9)	20.6(8.4–32.8)

#### Uptake rate

The raw output from the mixed logit analysis model was used to estimate the predicted impact of various incentive packages on health workers’ job preferences. Each point estimate was compared against base levels in this study, which included the current salary (0% increase), no education, no housing, health facilities located two hours away from district centers or towns on par with district centers, unsupportive management, inadequate medicine and supplies, and insufficient infrastructure. While this baseline scenario may appear unfavorable, it closely reflects the conditions in rural health facilities in the South Omo zone. Although we predicted job uptake probabilities for each attribute level change, we report preference impact measures based on combinations of incentives, which health workers generally prefer over single incentives.

Table 6 presents the preference impact values for different attraction and retention intervention packages, across four levels of potential salary increase–0%, 40%, 60%, and 80%– as included in the DCE survey instrument. The packages are listed in order of highest to lowest preference. These predictions are valuable for policymakers, as they demonstrate how health workers’ decisions respond to variations in multiple job attributes and identify feasible combinations.

As indicated in Table 6, a combination of various incentive packages is generally favored over single incentives. For instance, a package that includes opportunities for education upgrading after one year of service, advanced housing, supportive management, timely salary payments, access to adequate medicine and supplies, and sufficient infrastructure, combined with 80% salary increase, was preferred by 97.2% of the respondents compared to their current situation. Table 6 also indicates that as the salary increases, the percentage of respondents favoring a given job over their current job increases for any incentive package. However, for packages with high preference impact at the current salary level, additional salary increases have a limited effect on preference. For example, package one is preferred by 94.3% of health workers with current salary, rising only to 97.2% with an 80% increase-yielding less than a 3% rise in preference, despite the high cost of an 80% salary increase. By contrast, for packages with lower initial preference impact, increasing salary has a larger impact on desirability. For example, package 5 has a preference impact of 87.9% at current salary, which rises by approximately 6% points with an 80% increase. This information is crucial for policymaking, as it demonstrates which job attribute combinations are most attractive to health workers and offers insight into politically and financially feasible attraction and retention strategies.

**Table 6:** Predicted Preference Impact of Retention Strategy Packages for Health Workers

Potential Retention Strategy	Health Workers			
**Salary Increase**	0%	40%	60%	80%
**Package One**				
Provide education opportunities for further study after 1 year	94.3%	96%	96.7%	97.2%
Provide advanced housing				
Management is supportive and make work easy				
Salary and other benefits paid on time				
Adequate medicine and equipment's				
Sufficient infrastructure				
**Package Two**				
Provide education opportunities for further study after 1 year	93.6%	95.5%	96.3%	96.9%
Provide advanced housing				
Management is supportive and make work easy				
Salary and other benefits paid on time				
Sufficient infrastructure				
**Package Three**				
Provide education opportunities for further study after 1 year	92.6%	94.8%	95.6%	96.3%
Provide advanced housing				
Management is supportive and make work easy				
Salary and other benefits paid on time				
**Package Four**				
Provide education opportunities for further study after 1 year	91.1%	93.7%	94.7%	95.6%
Provide advanced housing				
Management is supportive and make work easy				
**Package Five**				
Provide education opportunities for further study after 1 year	87.9%	91.3%	92.7%	93.9%
Provide advanced housing				
**Package Six**				
Provide education opportunities for further study after 1 year	79.8%	85.2%	87.4%	89.3%

## Discussion

Most discrete choice experiments (DCEs) analyze national-level data to determine health workers’ job location preferences across diverse rural areas, with findings that inform national policies aimed at improving health worker distribution in these regions [20, 22, 30]. Our study uses a DCE to examine the job preferences of health workers using subnational data in Ethiopia. We included attributes considered potential policy tools for attracting and retaining health workers in rural locations [6]. All eight attributes considered in this study were found to have a statistically significant influence on health workers’ preferences for healthcare jobs. Health workers preferred jobs offering higher salaries, education opportunities, advanced housing, timely payments, supportive management, adequate equipment and medicines, and sufficient infrastructure.

The findings from this study revealed that an improved salary had a statistically significant impact on the job choices of health workers in the South Omo zone. Currently, South Omo zone lacks such incentives, and salary levels in Ethiopia are notably low globally. As of March 2022, Ethiopia’s average monthly salary stands at $174 and, after recent devaluation, drops further to $74, the lowest in Africa [52]. Implementing an 80% salary increase could potentially address health worker shortages in areas largely populated by rural communities, aligning with previous studies in similar settings [19, 21]. For example, Robyn et al. conducted a DCE survey among students and health workers in Cameroon, finding that a rural retention bonus of 75% of the base salary was the attribute with the largest effect on attracting and retaining health workers in rural regions [21]. However, to attract health workers in rural areas, salaries must be significantly increased, as indicated by Kruk et al. [14] and Blaauw et al. [12].

In addition to salary, non-monetary factors also play an important role in influencing health workers’ decisions to relocate to or remain in predominantly rural regions. We found that education upgrading opportunities were the strongest driver of job choice among health workers. FGDs and in-depth interviews with health workers revealed that these opportunities were key factors in their decision to work in rural areas –findings supported by our DCE results, which showed education upgrading as the most valued job attribute based on WTP estimates. This aspiration for educational opportunities likely stems from the potential for salary increases and access to prestigious positions. Incorporating these opportunities after a designated service period in rural areas could address the imbalance in health worker distribution between urban and rural areas. However, these opportunities should be tied to legally binding contracts requiring post-graduation service in rural areas to prevent worker loss to cities and non-governmental organizations. These results align with findings from other DCEs conducted in low- and middle-income countries [20, 53, 54].

Among non-monetary attributes, the provision of housing emerges as the second most crucial job factor influencing health workers’ decisions to relocate to or stay in rural positions. This finding is very intuitive, as rural areas offer minimal or no rental housing, and available housing in small towns is often unaffordable. The lack of housing is frequently cited as a barrier to relocation or retention in rural areas, as noted by health workforce during in-depth interviews and FGDs. Except for two health facilities constructed by non-governmental organizations (which are not part of this study), all government constructed health centers lack housing for health workers. In the South Omo zone, only two out of the 25 surveyed health centers are located in small towns where very few rental housings are available. However, even these options are often costly, and many lack privacy and autonomy due to the absence of rental regulations in small towns. In remote areas, housing is typically unavailable because pastoral communities do not build permanent structures. As a result, many health workers repurpose parts of health facilities’ buildings as living and cooking spaces, which can impact the quality of health services.

The high preference for housing, therefore, may arise from health workers limited ability to afford house construction, the lack of rental housing in remote locations, and the fact that rent in small towns often increases more rapidly than health worker salaries. Moreover, government provided houses could offer greater privacy and autonomy compared to private rentals. Therefore, to retain health workers and ensure high quality health services, policymakers should prioritize implementing housing interventions in rural areas. The finding that housing provision statistically significantly influences health workers’ decisions aligns with earlier DCE studies [23, 24]. For instance, Berman et al. conducted a DCE survey among final-year college students and practicing nurse-midwifery technicians in Malawi and found that housing provision was a key factor in decisions to relocate to or remain in rural locations.

Health workers were more likely to select jobs with a supportive management system at the workplace, as evidenced by both qualitative and DCE studies [14, 24, 29, 55]. In this study, supportive management refers to the positive relationships among health workers and between health workers and their supervisors, including health center heads and district officials. Supportive management is a crucial factor influencing health workers’ job satisfaction and decisions regarding rural postings [14, 55]. Through in-depth interviews and focus group discussions, health workers expressed a desire for supportive management at health facilities and district health offices to encourage them to go to or remain in rural postings. Specific points raised by health workers included the need for regular supportive supervision and constructive feedback. This finding is supported by studies conducted in other developing countries [56, 57].

A lack of empathy and understanding from supervisors was identified as a significant reason for job dissatisfaction and high turnover in rural postings in Ethiopia [29]. Human resource managers’ recognition of the importance of fostering positive relationships between managers and health workers is crucial, as these relationships significantly affect the attraction and retention of health workers in rural settings [14]. Similar findings were observed in other DCE studies conducted among health workers and health science college students in low- and middle-income countries [14, 30, 41]. For example, a DCE study conducted in Ghana among 302 students identified supportive management as one of the key factors to attract them in rural areas [24]. Therefore, policymakers should consider supportive management as part of an incentive package to attract and retain health workers in rural areas, particularly in southwestern Ethiopia.

We also found that timely payment of salaries and benefits is highly valued by health workers. However, the government has faced significant challenges, including ongoing conflicts, which have affected the regular disbursement of funds. Ethiopia’s government budget has increasingly prioritized military spending over social and economic sectors in the last five years, resulting in dwindling allocations for essential services [58]. This has led to difficulties in ensuring on-time payment of health workers’ salaries and benefits, contributing to dissatisfaction and turnover in the South Omo zone. The situation is exacerbated by Ethiopia’s low salaries, which are among the lowest in Africa, and rapidly increasing inflation, which severely impacts families’ welfare when payment is delayed [52]. In-depth interviews, focus group discussions, and the 2022 South Omo zone health department annual review meeting (attended by the corresponding author) consistently highlighted payment delays as a top concern among health workers. Therefore, to enhance the appeal of rural health jobs, zonal and regional administrations must prioritize timely payment of salaries and benefits, particularly for health workers serving in rural areas.

The availability of equipment and medicine at health facilities has a limited impact on job selection in our study, which contrasts with findings from other DCEs in Tanzania [20], Malawi [24], and Ghana [14], where this attribute had a larger impact. Although providing equipment and medicine is relatively feasible for local and state governments, health workers may doubt the likelihood of actual implementation due to issues outside the scope of health system leadership (e.g., foreign currency shortage for importing medicine). However, incorporating this attribute into an incentive package could still improve the attraction and retention of health workers in rural areas. Similarly, infrastructure appears to have minimal impact on job selection among health workers in our study, which deviates from results found in other studies [14, 20]. Health workers may either doubt health system leadership’s capacity to improve infrastructure or prioritize other job aspects over infrastructure. Nevertheless, including infrastructure as part of an incentive package could improve the attractiveness of rural healthcare jobs.

The World Health Organization (WHO) recommends that bundling multiple incentives is the most effective strategy for attracting and retaining health workers in rural areas [59]. This study found that combining highest levels of salary, education opportunity, housing, timelines of payment, adequate medicine and supplies, and infrastructure attracted 97.2% of survey participants to rural positions. Consistent with similar studies, this bundled approach can significantly increase the likelihood of rural job acceptance [21, 22]. For example, in a DCE study in Laos, Jaskiewicz and his team found that combining the highest incentives levels for each attribute resulted in a job that was favored by nearly 98% of those surveyed [23].

We recognize there are limitations to this study. First, while the included attributes reflect health workers’ preferences as identified through literature review, IDIs, and FGDs, there may still be important factors not captured in this study. Second, although studies show that DCEs can effectively predict actual behavior in the public health sector [41], practical testing of the impact of the identified incentives would provide a better understanding of their actual impact. Finally, while this analysis addresses the impact of implementing policy measures, it does not include the costs associated with them. Implementing the recommended incentive package depends on the political and financial feasibility of these measures, therefore making a cost analysis an essential next step.

## Conclusion

Addressing the geographical imbalance in the distribution of health workers and ensuring the provision of essential health services in underserved regions requires an understanding of the factors influencing health workers’ decisions to relocate to or remain in rural areas. This study uses a discrete choice experiment to investigate the job preferences of health workers in the South Omo zone. Descriptive analysis revealed that most health workers are willing to relocate to or continue serving in rural areas, contingent upon improved working conditions. The data from the discrete choice experiment highlight both monetary and non-monetary incentives as effective means to attract and retain health workers in rural areas of South Omo zone. Furthermore, bundling incentives proved to be more effective than single incentives in influencing health workers’ decision to relocate to or remain in rural postings. However, during interviews and focus group discussions, health workers expressed doubts about the feasibility of implementing these incentives, citing a lack of trust in human resource managers’ ability to deliver on promises. To address this and improve the geographical distribution of the health workforce, health systems should consider offering legally binding agreements to ensure that incentive packages are honored, thereby increasing health workers’ confidence to take up or remain in rural positions.

## Supplementary Information

Supplementary Material 1.

Supplementary Material 2.

## Data Availability

Data is available upon request. Abdela Hilo can be contacted at banati1978@gmail.com.

## Footnotes

1 The data collection team consisted of the lead author, a driver, and a public health officer proficient in multiple local languages.

2 A large share of the income for health workers comes from outside of their salary in the form of per diem and other income from NGOs and other sources who work through the local health workers.

3 Defined as ‘electricity, water, transportation, & internet’.
