# Supplementary material for: Addressing concerns of access and distribution of health workforce: a discrete choice experiment to develop rural attraction and retention strategies in southwestern Ethiopia
Source: BMC Health Serv Res. 2024 Dec 18;24:1603. doi: 10.1186/s12913-024-11971-4 (PMC11654134; doi:10.1186/s12913-024-11971-4)
Supplement: Supplementary file 1 — Supplementary Material 1. [file 12913_2024_11971_MOESM1_ESM.pdf]

Protocol Title: Policy Interventions to Attract and Retain Health Workers to Rural and Remote Districts of South Ethiopia: Discrete Choice Experiment.

Abdela Alte Hilo

Qualitative Interview Questions for Health Workers[1]

The qualitative interview questions are prepared to explore health workers' motivations for continually working in rural areas and students' motivations to go to work in rural and remote areas. The qualitative interview questions should only be asked after the participant has provided informed consent. If participants seem uncomfortable responding to any of the questions, you can remind them that their answers are confidential, but also let them know they do not have to respond if they do not want to.

Familiarization Questions

1. I would like to learn about your background, tell me a little bit about yourself:
    a. In which college/university did you study to become a health professional?
    b. Where did you grow up? Which State/Zone/District? Was it in a town, was it a pastoral area or in a rural agrarian area?
2. Tell me about your family.
    a. Are you married? Are you living with your spouse? How big is your family? Do you have children? How many? Are your children living with you?
    b. Are you deeply religious? Do you have a religious organization in your place of work? Do you have a good relationship with your church/mosque community?

Work-Related Questions

1. Why did you choose to become a health professional? What are the things that motivated you to become a health professional?
    a. Are those factors still important to you now?
    b. Are you getting all the things you expected to get from the profession when you decided to become a health professional?
2. How long have you worked at this health facility?
    a. Have you worked at other health facilities before working at this facility?
    b. Have you ever imagined yourself in another facilities? Where? Why?
3. Tell me what you like about your job.
    a. Do you think that other health workers enjoy similar things about their jobs? Why?
4. Are there things you wish could change about your job that can increase health workers satisfaction and retention in rural South Omo?
    a. What are they?
    b. What would it take to make that change happen? Who would have to be responsible for it? Do you think it is possible? How?

Questions about Location Preference and Job Attributes

[1] I used materials from DCE guideline (WHO & WB, 2012), from Uganda DCE by (Rockers et al., 2012), and from Malawi DCE by (Berman et al., 2021) to develop the questions.

1. South Omo zone has a wide range of livelihood systems (i.e., pure pastoral to agro-pastoral and agrarian communities), have different ethnic groups, distinct levels of urban and rural locations, and varying climate conditions. What type of community or which climate condition would you most like to work in?

    a. Why do you prefer specific livelihood system or climate condition?
    b. Do you think working in pastoral woredas has advantages?
    c. Do you think working in agrarian woredas has advantages?
    d. What are disadvantages of working in pastoral woredas?
    e. What are disadvantages of working in agrarian woredas?

2. Thinking in general about the possibility of working in rural facilities, what are the most crucial factors that influence your decision to work continuously in a rural area? List them. (Note: facilitators register the list and make participants think more about other factors).
3. Please rank the following attributes in the order of priority for you to work in rural health facility (if they have factors other than those in the table facilitators will add them in the open spot). Provide an account of why they ranked the attributes as they did.

**Attributes**	**Rank**
Above Average Salary	
Education Opportunities/Upgrading After Service	
Availability Infrastructure (Internet, electricity, water, & transportation)	
Provision of Quality Housing	
Location	
Workload	
Positive Relationship with Peers and High-Level Supervisors	
Good Education for Children	
Timely Payment of Salary and Overtime Wages	
High Quality Health Center with Necessary Equipment's and Adequate Medicine	
Proximity to Towns and Family	
Shorter Compulsory Service Time	
Availability of Short-Term Training Opportunities	
Provision of Hardship Allowances	
Permission to have Private Wing in the Health Centers and Hospitals	

Focus Group Discussion Questions for Health Workers

The focus group discussion questions are prepared to explore health workers' and college students' drives for working in rural areas. These questions can only be asked after the participant has provided informed consent. If participants seem uncomfortable answering any questions, facilitators remind them that their answers are confidential, but also let them know they do not have to respond if they do not want to.

1. How do you feel about health care job postings in Ethiopia? Have you worked in other sectors before becoming a health worker?
2. Do you have experience of working in rural areas? What do you like or not like about working in rural health facilities? Are there things you wish would change to make working in rural health facilities more comfortable? Do you think change is possible?
3. What are the crucial factors you consider shaping your decision process to remain in rural health facilities?
4. Participants will be asked questions about the following job attributes identified as potentially important, based on the literature review, and asked to add to the list if there is any missing?

**Attributes**	**Rank**
Above Average Salary	
Education Opportunities/Upgrading After Service	
Availability Infrastructure (Internet, electricity, water, & transportation)	
Provision of Quality Housing	
Location	
Workload	
Positive Relationship with Peers and High-Level Supervisors	
Good Education for Children	
Timely Payment of Salary and Overtime Wages	
High Quality Health Center with Necessary Equipment’s and Adequate Medicine	
Proximity to Towns and Family	
Shorter Compulsory Service Time	
Availability of Short-Term Training Opportunities	
Provision of Hardship Allowances	
Permission to have Private Wing in the Health Centers and Hospitals	

5. Health workers will be asked to rank attributes according to what they believe to be the most important to them.
6. For the top eight ranked attributes, the participants will be asked to identify a range of realistic levels.

Attributes	Levels
Above Average Salary	
Education Opportunities/Upgrading After Service	
Availability Infrastructure (Internet, electricity, water, & transportation)	
Provision of Quality Housing	
Location	
Workload	
Positive Relationship with Peers and High-Level Supervisors	
Good Education for Children	
Timely Payment of Salary and Overtime Wages	
High Quality Health Center with Necessary Equipment’s and Adequate Medicine	
Proximity to Towns and Family	
Shorter Compulsory Service Time	
Availability of Short-Term Training Opportunities	
Provision of Hardship Allowances	
Permission to have Private Wing in the Health Centers and Hospitals	
