# Supplementary material for: Addressing concerns of access and distribution of health workforce: a discrete choice experiment to develop rural attraction and retention strategies in southwestern Ethiopia
Source: BMC Health Serv Res. 2024 Dec 18;24:1603. doi: 10.1186/s12913-024-11971-4 (PMC11654134; doi:10.1186/s12913-024-11971-4)
Supplement: Supplementary file 2 — Supplementary Material 2. [file 12913_2024_11971_MOESM2_ESM.docx]

Appendix

Table S1. In-depth interview ranking of health workers.

| Attribute | 15 | 14 | 13 | 12 | 11 | 10 | 9 | 8 | 7 | 6 | 5 | 4 | 3 | 2 | 1 | Total | Rank |
| --- | --- | --- | --- | --- | --- | --- | --- | --- | --- | --- | --- | --- | --- | --- | --- | --- | --- |
| **Infrastructure** |  | **1** |  |  |  |  |  |  | **2** |  | **2** |  |  | **3** | **8** | **52** | **1** |
| **Education opportunity** |  |  |  |  |  |  |  |  | **2** | **1** |  | **3** | **7** | **5** |  | **63** | **2** |
| **Timely Payment** |  |  | **1** |  | **2** |  |  |  |  | **1** | **1** | **4** | **3** | **3** | **4** | **81** | **3** |
| **Location** |  | **2** |  |  | **1** |  |  | **1** | **2** | **3** |  | **1** | **1** | **1** | **4** | **92** | **4** |
| **Salary** |  | **2** |  | **1** |  |  | **1** | **1** | **1** | **3** |  | **3** | **1** | **1** | **2** | **101** | **5** |
| **Adequate Equipment** |  | **1** |  | **1** |  | **1** | **1** |  | **2** |  | **3** | **5** | **1** | **3** | **1** | **104** | **6** |
| **Housing** |  |  |  | **1** | **1** | **1** | **2** | **2** | **1** | **3** | **2** | **1** |  | **2** |  | **110** | **7** |
| **Management Relationship** |  | **2** |  |  |  | **2** |  | **5** | **1** | **1** | **2** | **1** | **2** |  |  | **121** | **8** |
| Short-term training | 2 | 1 |  |  |  |  | 3 | 3 | 1 | 2 | 1 |  | 2 |  |  | 125 | 9 |
| Proximity to Towns | 1 |  | 1 | 3 | 2 | 2 | 3 | 1 |  |  | 2 |  |  |  |  | 131 | 10 |
| Workload | 2 |  |  |  | 3 | 2 | 1 | 1 | 2 | 2 | 1 | 1 |  |  |  | 139 | 11 |
| Having Private Wing | 1 | 2 | 4 | 2 |  | 2 |  |  |  | 1 | 1 |  |  |  |  | 150 | 12 |
| Hardship Allowances |  |  | 2 | 1 | 4 | 2 | 3 | 1 | 1 |  | 2 |  |  |  |  | 154 | 13 |
| Good Education for Children | 5 |  | 2 | 2 | 1 |  |  | 2 |  | 2 |  |  |  | 1 |  | 166 | 14 |
| Shorter compulsory service | 2 |  | 3 | 3 | 1 | 2 | 2 | 1 | 1 |  |  |  | 1 |  |  | 172 | 15 |
